# CRISPR/Cas9 Essential Gene Editing in Drosophila

**DOI:** 10.32607/actanaturae.11874

**Published:** 2023

**Authors:** I. S. Osadchiy, S. O. Kamalyan, K. Y. Tumashova, P. G. Georgiev, O. G. Maksimenko

**Affiliations:** Institute of Gene Biology, Russian Academy of Sciences, Moscow, 119334 Russian Federation

**Keywords:** CRISPR/Cas9, genome editing, essential gene editing, housekeeping genes

## Abstract

Since the addition of the CRISPR/Cas9 technology to the genetic engineering toolbox, the problems of low efficiency and off-target effects hamper its widespread use in all fields of life sciences. Furthermore, essential gene knockout usually results in failure and it is often not obvious whether the gene of interest is an essential one. Here, we report on a new strategy to improve the CRISPR/Cas9 genome editing, which is based on the idea that editing efficiency is tightly linked to how essential the gene to be modified is. The more essential the gene, the less the efficiency of the editing and the larger the number of off-targets, due to the survivorship bias. Considering this, we generated deletions of three essential genes in *Drosophila*: *trf2, top2, *and *mep-1*, using fly strains with previous target gene overexpression (“pre-rescued” genetic background).

## INTRODUCTION

Recent advances in the use of CRISPR/Cas9 as a programmable tool for the introduction of DNA double- strand breaks significantly expanded possibilities in deciphering the functions of genes and genomic regulatory elements. The CRISPR/Cas9 system is the most suitable for knocking out a gene of interest (GOI) by generating shifts in the reading frame of the target gene. However, if the GOI is an essential one, attempts to generate a knock-out can be ineffective due to lethality in successfully edited embryos, biological plasticity that rescues the induced frameshift or deletion by translation reinitiation, defective exon skipping, etc. [1]. Here, we report on a case of CRISPR/Cas9 use, in combination with target gene overexpression, that allowed us to quite effectively knock out three essential genes in *Drosophila*. A similar approach has recently been validated in human HEK293T cells [2]. By using this approach, we deleted a relatively long region of the GOI coding sequence and replaced it with a landing platform, which allows for fast and effective insertion of modified gene constructs.

## EXPERIMENTAL

**Fig. 1:**
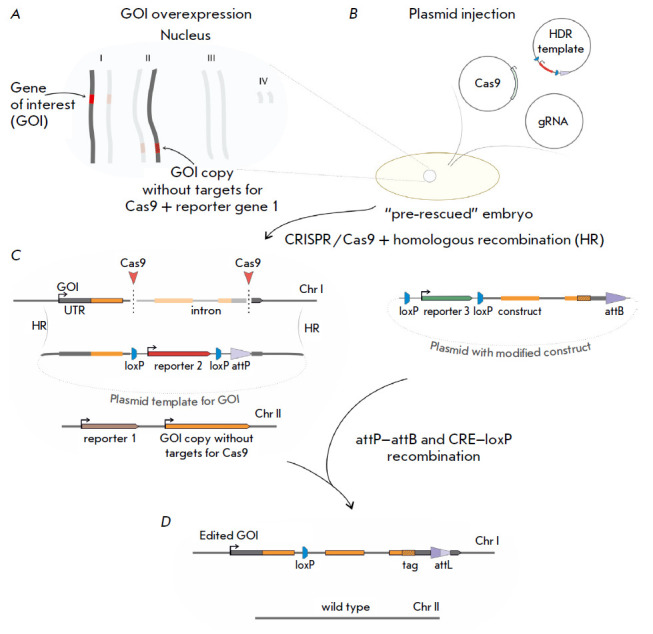
The strategy for essential gene replacement. (*A*) Insertion of a gene copy lacking targets for Cas9 and reporter gene 1 (*yellow*) into a “safe harbor” knock-in site on a different chromosome via site-specific recombinase-mediated integration (SSRMI). (*B*) Microinjection of an HDR template and plasmids expressing Cas9 and gRNAs into “pre-rescued” embryos. (*C*) CRISPR/Cas9-mediated DNA double-strand breaks and homologous recombination (HR) with the plasmid template carrying *loxP*-flanked reporter gene 2 (*mCherry*) and an *attP *site. (*D*) The result of subsequent SSRMI of the modified gene of interest (GOI) sequence followed by CRE-mediated reporter gene 2 (*mCherry*) and 3 (*white*) excision and removal of the GOI copy

The strategy presented here is an addition to the methods described in [3, 4, 5] and suitable for ubiquitously expressed essential genes. Our method consists of three steps (*Fig. 1*):

1. Insertion of the GOI copy (lacking CRISPR/Cas9 target sequences) and reporter gene 1 into a “safe harbor” knock-in site located on a different chromosome. This step results in the generation of the rescue line with homologous expression of the GOI copy. For this, we have created rescue constructs carrying protein- coding sequences of either TRF2, Top2, or MEP-1 under the control of the *Ubi-p63E *promoter and the* yellow *gene as reporter 1. Previously obtained knockouts of these genes were embryonic lethal. The constructs were inserted into either 86Fb (TRF2/Top2) or 38D (MEP-1) chromosomal loci via φC31-mediated site-specific integration.

2. Replacement of the GOI with the *attP *site by injection of three plasmids: carrying Cas9 and gRNAs for extensive deletion of the GOI protein-coding sequence and a template plasmid for homology-directed repair (HDR) containing the *attP *site for the φC31 integrase and reporter gene 2 (*mCherry*), flanked by* loxP *sites. This step results in the generation of the GOI knockout line with a GOI copy overexpression background.

In this work, the following CRISPR/Cas9* Drosophila *strains obtained from The Bloomington* Drosophila *Stock Center at Indiana University were used: BL54591 (*Cas9 *under the control of the *nanos* promoter) and BL58492 (*Cas9 *under the control of the *Actin5C *promoter). Alternatively, the Addgene #62209 helper plasmid was added to the injection mixture as a source of Cas9. CRISPR targets were designed using the Optimal Target Finder software (University of Wisconsin) [4] and cloned into the vector based on pCFD4-U6:1_U6:3tandemgRNAs (Addgene #49411). The following gRNAs were used for *trf2 *deletion: gRNA1 (tcttcgtgcatactcttagc), gRNA2 (tgcttttcgcttcggtgtcc), and gRNA3 (accaagtagctagagactta); the gRNA1/gRNA2 pair leads to deletion of a 6.7 kb genomic fragment; gRNA1/gRNA3 causes deletion of a 1.1 kb fragment. For *mep-1, *the following gRNAs were used: gRNA1m (acgaacagcagggcgcgcgc), gRNA2m (cagcaagtgacgctggcttg), and gRNA3m (aggggatcttcggcctcgca). They produce 5.6 (gRNA1m/ gRNA2m) and 2 kb (gRNA1m/ gRNA3m) deletions. For *top2 *deletion, gRNA1t (gttcccagtacagtagcacc) and gRNA2t (tctacggcgtgttcccgctt) producing a 2 kb deletion were used.

The flies obtained after injection (F0) were individually mated with *y1w1118 *flies; potential genome editing events in the progeny (F1) were detected by mCherry fluorescence. The insertion of the landing platform (attP-mCherry) into the genome was confirmed by PCR with primers annealing outside the homology regions used for HDR.

3. Insertion of a modified GOI variant labelled with loxP-flanked reporter gene 3 (*white *gene) via sitespecific recombination. Flies were injected with a mixture containing two plasmids: a plasmid with a modified gene variant and the attB site, and the φC31 integrase helper plasmid (Addgene #26290). After integration of the modified variant, reporter genes 2 and 3 were removed by crossing with a Cre recombinase- expressing line.

## RESULTS AND DISCUSSION

The TRF2 protein is a paralog of the basal transcription factor TBP; its inactivation is associated with embryonic lethality [6, 7].

**Fig. 2:**
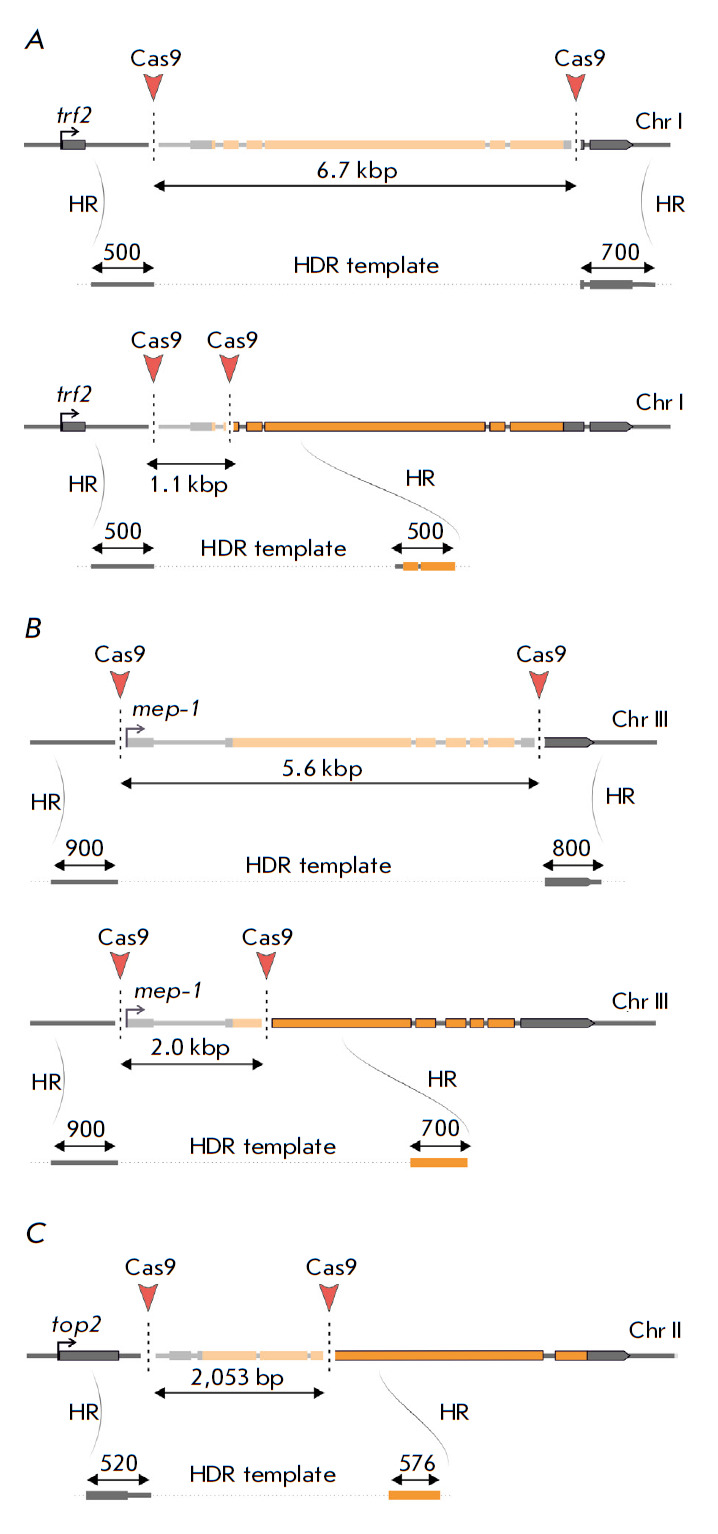
CRISPR/Cas9- and HDR-mediated gene replacement with the *attP *site and reporter gene *mCherry*. The genes *trf2 *(*A*), *mep-1 *(*B*), and *top2 *(*C*) and homologous recombination templates for either full-length or partial deletions are presented

Previously, we failed to replace the *trf2 *gene with a landing platform for site-specific integration of modified gene variants despite the use of two different Cas9 sources (Cas9-expressing fly lines and the Cas9- expressing plasmid injected into embryos) and two gRNA combinations [8]. The whole *trf2 *gene spans approximately 25 kb, while its protein-coding region is roughly 7 kb. The chosen gRNA combinations produced two DNA double-strand breaks at distances of 6.7 and 1.1 kb for deletion and concomitant replacement by the landing platform of the whole proteincoding region or only the start codon-containing region, respectively (*Fig. 2A*).

The results obtained for the different editing schemes used for *trf2 *gene replacement are summarized in *Table 1*.

**Table 1:** Results of plasmid microinjections for the replacement of the trf2, mep-1, and top2 genomic regions with a landing platform

Fly line		Cas9 source	Deletion, bp	Embryos injected	Flies eclosed, F0	mCherry+ F1 lines	Off-targets
TRF2	y^1^w^1118^	Cas9-expressing plasmid	6700	200	100	–	–
	54591	Cas9 under nanos promoter	6700	250	140	1	+
	58492	Cas9 under Actin5C promoter	6700	200	80	–	–
			1100	250	120	–	–
	y^1^w^1118^ + TRF2 overexpression	Cas9-expressing plasmid	6700	100	80	5	2
			1100	100	80	5	2
MEP-1	y^1^w^1118^	Cas9-expressing plasmid	2000	300	160	–	–
			5600	150	90	1	–
	y^1^w^1118^ + MEP-1 overexpression		5600	240	175	4	–
TOP2	y^1^w^1118^	Cas9-expressing plasmid	2053	150	100	–	–
	y^1^w^1118^ + MEP-1 overexpression		2053	150	80	3	–

The F0 embryos without background *trf2 *overexpression were characterized by a low survival rate. In the developing larvae, *mCherry *reporter fluorescence was observed in tissues in the vicinity of the injection site and throughout the whole embryo. The larvae with the most spread and intense fluorescence died later during development. As a result of mating the surviving F0 flies with the wild-type line, only one fly line with insertion of the landing platform into the intron corresponding to the 5` double-strand break without the deletion of the *trf2 *coding region was obtained.

In order to overcome the high lethality rate due to* trf2 *deletion, we generated a fly line with *trf2 *overexpression by site-specific integration of the *trf2 *short isoform using a line with the attP at locus 86Fb.

The *trf2*-overexpressing embryos injected with the gene editing mix had normal viability. As a result, we obtained five fly lines with insertion of the *mCherry* reporter gene for each of the gRNA combinations, producing 6.6 and 1.1 kb deletions, respectively.

We additionally validated this approach on other genes: *mep-1 *and *top2.*

MEP-1 is a protein that facilitates the recruitment of the nucleosome remodeling and histone deacetylation (dNuRD) complex to many gene promoters [9, 10]. It is an important regulator of early development in *Drosophila*; *mep-1 *gene inactivation leads to embryonic lethality.

As in the case of *trf2*, the selected gRNA combinations resulted in two DNA double-strand breaks spaced 5.6 or 2 kb apart for the full-length and start codon region deletions, respectively (*Fig. 2B*). The results obtained for the different editing schemes used for *mep-1 *gene replacement are summarized in *Table 1*.

Embryos injected with the mixture for *mep-1 *gene replacement without *mep-1 *overexpression background had moderate lethality during development. Mating of F0 flies resulted in only one fly line, which had a long gene deletion. Meanwhile, injection of the embryos with background *mep-1 *overexpression led to the generation of four fly lines with the landing platform. Thus, *mep-1 *deletion is not completely lethal; however, its overexpression increases the viability of injected embryos and, as a consequence, gene editing effectiveness.

Topoisomerase 2 (Top2) is an enzyme that releases topological tension in the DNA molecule; it contributes to genome stability and participates in key cell processes such as replication, transcription, and recombination [11].

For the replacement of the *top2 *gene with the landing platform, we designed a pair of gRNAs targeting Cas9 to the loci 2 kb apart from each other located in 5`UTR and exon 3 of *top2*. The editing plasmid mixture for gene replacement was injected into* y1w1118 *fly embryos. There were no cases of platform insertion in the progeny of individual matings of F0 with wild type flies. However, editing upon insertion of the Top2 coding sequence in the 86Fb chromosomal locus resulted in three knockout fly lines (*Table 1*).

The use of Cas9 for genome editing is frequently accompanied by additional unspecified mutations throughout the genome. Since mutations usually manifest themselves through phenotype and/or a change in the survival rate, GOI overexpression on a different chromosome allows one to probe the mutations on the GOI chromosome in a line homozygous for GOI deletion. Therefore, it is possible to select only lines without severe mutations.

The generated fly lines homozygous for Δ*trf2*, Δ*mep-1, *or Δ*top2 *deletion were lethal without the additional rescuing copy*. *This corroborated the essentiality of the edited genes and provided initial evidence of successful gene replacement with the *attP*-platform. Site-specific integration of a restoring construct (coding for the wild-type gene variant) into the corresponding landing platform line and subsequent removal of the reporter genes led to the recovery of gene function and normal viability of homozygous flies lacking the rescuing copy. Thus, overexpression induced prior to gene editing allowed us to obtain landing platforms for a detailed study of three* Drosophila *proteins: TRF2, Top2, and Mep-1.

## Abbreviations

gRNA: guide RNA
chr: chromosome
kb: kilobase
TRF2: TBP-related factor 2
Top2: type II topoisomerase
MEP-1: MOG interacting and ectopic P-granules protein 1
